# Reduced growth velocity from the mid-trimester is associated with placental insufficiency in fetuses born at a normal birthweight

**DOI:** 10.1186/s12916-020-01869-3

**Published:** 2020-12-24

**Authors:** Lucy M. Kennedy, Stephen Tong, Alice J. Robinson, Richard J. Hiscock, Lisa Hui, Kirsten M. Dane, Anna L. Middleton, Susan P. Walker, Teresa M. MacDonald

**Affiliations:** 1 Department of Obstetrics and Gynaecology, University of Melbourne, Mercy Hospital for Women, 163 Studley Road, Heidelberg, VIC 3084 Australia; 2 Mercy Perinatal, Mercy Hospital for Women, Melbourne, VIC Australia

**Keywords:** Appropriate-for-gestational-age, Fetal growth restriction, Growth velocity, Mid-trimester, Placental insufficiency, Antenatal ultrasonography, Small-for-gestational-age

## Abstract

**Background:**

Fetal growth restriction (FGR) due to placental insufficiency is a major risk factor for stillbirth. While small-for-gestational-age (SGA; weight < 10th centile) is a commonly used proxy for FGR, detection of FGR among appropriate-for-gestational-age (AGA; weight ≥ 10th centile) fetuses remains an unmet need in clinical care. We aimed to determine whether reduced antenatal growth velocity from the time of routine mid-trimester ultrasound is associated with antenatal, intrapartum and postnatal indicators of placental insufficiency among term AGA infants.

**Methods:**

Three hundred and five women had biometry measurements recorded from their routine mid-trimester (20-week) ultrasound, at 28 and 36 weeks’ gestation, and delivered an AGA infant. Mid-trimester, 28- and 36-week estimated fetal weight (EFW) and abdominal circumference (AC) centiles were calculated. The EFW and AC growth velocities between 20 and 28 weeks, and 20–36 weeks, were examined as predictors of four clinical indicators of placental insufficiency: (i) low 36-week cerebroplacental ratio (CPR; CPR < 5th centile reflects cerebral redistribution—a fetal adaptation to hypoxia), (ii) neonatal acidosis (umbilical artery pH < 7.15) after the hypoxic challenge of labour, (iii) low neonatal body fat percentage (BF%) reflecting reduced nutritional reserve and (iv) placental weight < 10th centile.

**Results:**

Declining 20–36-week fetal growth velocity was associated with all indicators of placental insufficiency. Each one centile reduction in EFW between 20 and 36 weeks increased the odds of cerebral redistribution by 2.5% (odds ratio (OR) = 1.025, *P* = 0.001), the odds of neonatal acidosis by 2.7% (OR = 1.027, *P* = 0.002) and the odds of a < 10th centile placenta by 3.0% (OR = 1.030, *P* < 0.0001). Each one centile reduction in AC between 20 and 36 weeks increased the odds of neonatal acidosis by 3.1% (OR = 1.031, *P* = 0.0005), the odds of low neonatal BF% by 2.8% (OR = 1.028, *P* = 0.04) and the odds of placenta < 10th centile by 2.1% (OR = 1.021, *P* = 0.0004). Falls in EFW or AC of > 30 centiles between 20 and 36 weeks were associated with two–threefold increased relative risks of these indicators of placental insufficiency, while low 20–28-week growth velocities were not.

**Conclusions:**

Reduced growth velocity between 20 and 36 weeks among AGA fetuses is associated with antenatal, intrapartum and postnatal indicators of placental insufficiency. These fetuses potentially represent an important, under-recognised cohort at increased risk of stillbirth. Encouragingly, this novel fetal assessment would require only one additional ultrasound to current routine care, and adds to the potential benefits of routine 36-week ultrasound.

## Background

Fetal growth restriction (FGR) defines a fetus that fails to meet its biological growth potential. In high-income countries, FGR due to placental insufficiency is the most significant risk factor for stillbirth in normally formed fetuses [1, 2]. Importantly, when FGR remains undetected antenatally, the risk of stillbirth is increased approximately fourfold and is double the risk of detected FGR [1].

Small-for-gestational-age (SGA), defined as ultrasound estimated fetal weight (EFW), abdominal circumference (AC) or infant birthweight below the 10th centile, is the most commonly used proxy for FGR [3]. Its use as a surrogate for FGR has limitations, as SGA captures a heterogeneous population of both constitutionally (small but healthy) and pathologically, small fetuses [4]. Further, the use of the 10th centile as an arbitrary cut-off fails to recognise those appropriate-for-gestational-age (AGA) fetuses and neonates who have also experienced placental insufficiency. Such infants may have failed to achieve their growth potential, despite having a birthweight above the 10th centile for their gestational age. Significantly, 70% of stillbirths occurring at term occur in fetuses classified as AGA [5, 6]. These AGA infants are likely to have experienced late-onset FGR. The size of the fetus is affected to a lesser extent than in early onset FGR, as fetal nutritional demands plateau while oxygen demands increase [7]. If identified, timely delivery could prevent stillbirth in these cases. However, late-onset FGR is more difficult to detect antenatally [8], requiring a comprehensive approach that may incorporate assessment of both fetal size and growth [4, 9, 10].

We have previously shown that AGA infants who demonstrate slowing of EFW and AC growth velocities between 28 and 36 weeks’ gestation have increased rates of antenatal, intrapartum and neonatal outcomes indicative of placental insufficiency [11]. We propose that this ‘slowing growth’ population represents an at-risk group of fetuses, not traditionally identified as growth restricted. Detecting reduced antenatal growth velocity across the third trimester could potentially reduce stillbirth risk, through increased surveillance and timely delivery. However, such an approach requires the expensive and logistically challenging addition of two ultrasound scans to what currently constitutes standard care. The Pregnancy Outcome Prediction study reported that abdominal circumference growth velocity between 20 and 36 weeks’ gestation predicted adverse outcome among fetuses identified as SGA (EFW < 10th centile) [12]. Therefore, we wished to evaluate whether reduced growth velocity measured from the mid-trimester is also associated with indicators of placental insufficiency among the AGA cohort (birthweight > 10th centile). If so, this growth velocity assessment could be achieved with the addition of only one ultrasound, at 36 weeks’ gestation.

We also wished to identify the gestational epoch where falling growth velocity is most strongly associated with placental insufficiency. Therefore, we performed this study to examine the associations of 20–28 and 20–36-week growth velocities with measures of placental function and to compare these to our previously reported associations with 28–36-week growth velocities [11]. A comparison of growth velocities across these three different gestational epochs has not previously been made.

## Methods

### Study design overview

This study aimed to establish whether growth velocities in AGA fetuses best reflect placental function if measured between 20–28, 28–36, and 20–36 weeks’ gestation. We analysed data from participants of the Fetal Longitudinal Assessment of Growth (FLAG) study—a prospective cohort study conducted at a tertiary maternity hospital in Melbourne, Australia, which has been previously described in detail [11]. In brief, the FLAG study prospectively recruited nulliparous women who underwent blinded ultrasound scans at 28 and 36 weeks’ gestation. From these scans, 28–36-week estimated fetal weight (EFW) and abdominal circumference (AC) growth velocities were calculated, and we have reported that growth velocity correlates with antenatal, intrapartum and postnatal clinical indicators of placental insufficiency among infants born AGA [11]. The FLAG study was approved by the Mercy Health Research Ethics Committee (Ethics Approval Number R14/12) and written informed consent was obtained from all participants.

For this study, second trimester fetal size was determined using biometry measurements reported at the participants’ routine mid-trimester morphology ultrasound scan. One hundred eighty-seven (53.9%) of these were performed at the Mercy Hospital for Women, with the remainder being performed by external medical imaging providers. Gestation-dependent centiles were assigned for both mid-trimester EFW and AC. EFW and AC centile changes between 20–28 and 20–36 weeks were calculated and examined for associations with the same antenatal, intrapartum and postnatal indicators of placental insufficiency. These correlations were then compared to those previously reported for fetal growth velocities between 28 and 36 weeks [11].

This study was designed to investigate whether slowed growth velocities from the mid-trimester are associated with features of placental insufficiency among AGA infants delivered at term. Therefore, SGA infants (customised birthweight < 10th centile) were excluded from  the main analyses.

### Assigning gestation-specific EFW and AC centiles

Three hundred forty-seven of the participants recruited to the FLAG study completed both 28- and 36-week research ultrasound examinations and were therefore eligible for inclusion. Biometric measurements (biparietal diameter, head circumference, AC and femur length) were obtained from the mid-trimester morphology ultrasound and the EFW was derived from the Hadlock equation using all four parameters or from the Shepard formula utilising three parameters for two cases where there were missing femur length measurements [13, 14]. The mid-trimester scans had been performed between 17^+6^ and 23^+0^ weeks’ gestation. Twenty-eight- and 36-week fetal biometry measurements were obtained as previously described [11].

As in the original FLAG study, gestation-specific mid-trimester ultrasound EFW centiles were customised using the GROW software (http://www.gestation.net/) [15]. The GROW software was used to adjust for maternal height, weight and parity, fetal sex and gestational age. We did not customise for maternal ethnicity as it is known to be inconsistently assigned among our patient population [16]. The GROW software only assigns customised centiles from a minimum of 20 weeks’ gestation. For the 39% of participants who had their mid-trimester scan prior to 20 weeks, we calculated the Hadlock EFW gestation-specific centile at the gestation of their mid-trimester scan, and then calculated the EFW at 20 weeks’ gestation that would correlate to that same Hadlock centile. We then used the extrapolated 20-week EFW to calculate the customised centile using the GROW software. Coefficients for the Australian application of GROW were informed by a local dataset. All AC centiles were assigned using the Chitty AC equation [17].

### Calculating fetal growth velocity

We calculated the change in EFW centile between 20 and 28 weeks, by subtracting the 20-week customised EFW centile from the 28-week customised EFW centile. Similarly, we calculated the change in EFW centile between 20 and 36 weeks by subtracting the 20-week EFW centile from the 36-week centile. The same process was used to calculate AC growth velocities for both time epochs. A fetus with a reduction in EFW or AC centile over the time period thus had a negative number to describe the growth velocity, a fetus with no change in centile had a velocity value of zero and a fetus with an increase in centile across gestation had a positive velocity value.

To standardise comparison of growth velocities across the cohort, the change in both EFW and AC centiles between ultrasounds were divided by the exact number of days between examinations, to create a centile change per day. For the 20–28-week velocities, we then multiplied this number by 56 to facilitate standardised comparison of centile change over exactly 8 weeks. For the 20–36-week velocities, we multiplied this number by 112 to compare change over exactly 16 weeks. Consistent with our previous study [11], we defined low growth velocity as a fall in EFW or AC of at least 30 centiles between the two ultrasound scans to allow for the maximal ultrasound error (approximately 15%) when estimating fetal weight.

### Clinical indicators of placental insufficiency

The outcomes used as antenatal, intrapartum and postnatal evidence of placental insufficiency among AGA fetuses were as follows: cerebroplacental ratio (CPR) < 5th centile at 36 weeks—reflecting increasing placental resistance and redistribution of blood flow to the brain—a known fetal adaptation to relative hypoxia [18], neonatal acidosis (umbilical artery pH < 7.15 at birth) following the hypoxic challenge of labour—reflecting reduced placental reserve [19] and low neonatal body fat percentage (BF%) measured by air displacement plethysmography (ADP)—reflecting reduced in utero nutritional reserve (BF% < 4.2% for males and < 5.8% for females) [20]. Where possible, study infants had ADP performed within the first 4 days after birth prior to their discharge from hospital. ADP assessments in our cohort were performed at a mean (standard deviation) 44.7 (12.1) hours of age. Untrimmed placentas were weighed in the birth suite and were classified as either < 10th centile or ≥ 10th centile adjusted for gestation at birth and infant sex [21]. A placenta < 10th centile for gestation and infant sex was considered a further indicator of placental insufficiency as smaller placentas are associated with FGR [22].

### Statistical analysis of growth velocities and clinical indicators of placental insufficiency

#### Antenatal clinical indicators of placental insufficiency

We examined the relationship between fetal growth velocity and antenatal evidence of placental insufficiency (the CPR, a measure of fetal adaptive behaviour) in three ways. First, linear regression was used to measure the correlations between EFW and AC growth velocities and 36-week gestation-specific CPR multiples of the median (MoM). Secondly, logistic regression was used to assess correlations between EFW and AC growth velocities and abnormal 36-week CPR (< 5th centile [23]). Last, if there was a significant relationship observed with logistic regression, we calculated relative risk (RR) and associated 95% confidence interval (CI) of low CPR at our pre-defined clinical threshold (− 30 centiles) of low growth velocity.

#### Intrapartum clinical indicators of placental insufficiency

The relationship between growth velocity and intrapartum evidence of placental insufficiency was examined using logistic regression to assess the relationships between EFW and AC growth velocities and umbilical artery pH < 7.15 among those who underwent labour. Participants delivered by elective caesarean section were excluded from this analysis. If there was a significant relationship observed with logistic regression, we calculated RR and associated 95% CI of intrapartum acidosis at the pre-defined dichotomous clinical threshold of low growth velocity, − 30 centiles.

#### Neonatal indicators of placental insufficiency

We examined the relationship between fetal growth velocity and neonatal evidence of placental insufficiency using the same three methodologies applied to 36-week CPR. First, linear regression assessed the correlations between growth velocities and three neonatal body composition measures: Ponderal Index (birthweight (g) × 100)/length^3^ (cm)), neonatal BF% estimated by skinfold measurements and ADP BF%. Then, using logistic regression, the relationships between EFW and AC growth velocities and low ADP BF% (defined as < 4.2% for males and < 5.8% for females, equating to more than one standard deviation below the mean [20]) and placental weight < 10th centile [21] were assessed. Finally, if a significant relationship was observed with logistic regression, we calculated RR and associated 95% CI of both low ADP BF%, and placental weight < 10th centile, at our pre-defined dichotomous clinical threshold of low growth velocity (− 30 centiles).

Statistical analysis was performed using GraphPad Prism version 8.00 for Windows (GraphPad Software, San Diego, CA, USA, http://www.graphpad.com/), R version 3.3.3 and Stata Statistical Software Release 16 (College Station, TX, USA).

## Results

**Fig. 1:**
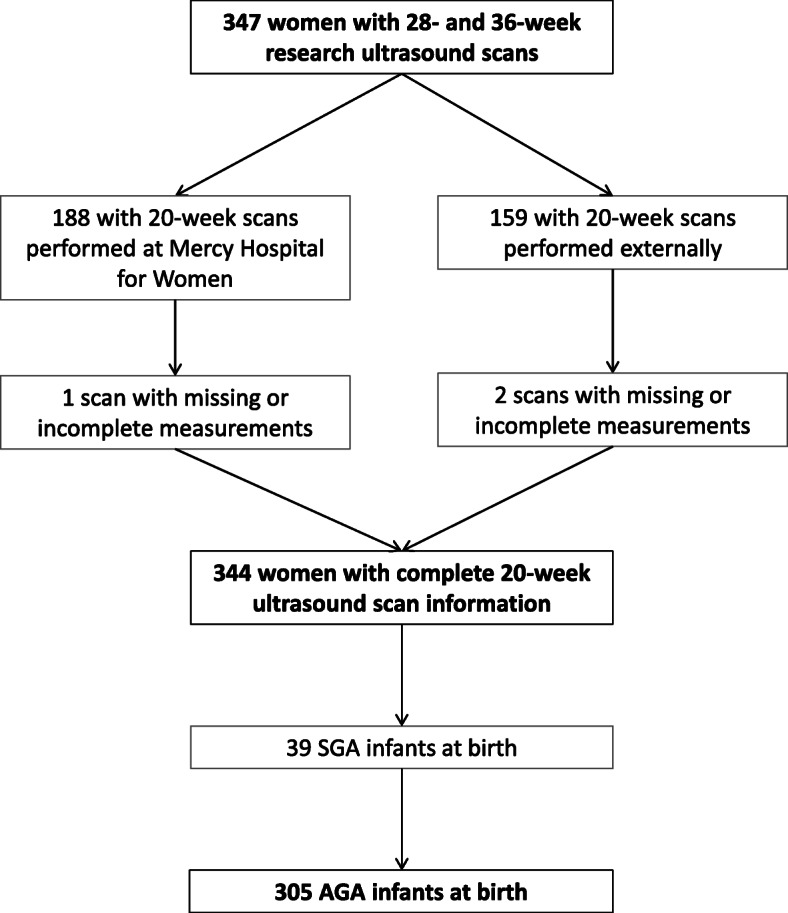
Study profile. AGA, appropriate-for-gestational-age (birthweight ≥ 10th centile); SGA, small-for-gestational-age (birthweight < 10th centile)

Three hundred forty-seven participants recruited to the FLAG study completed both 28- and 36-week ultrasound examinations. Three of these participants had missing or incomplete mid-trimester morphology scan reports, leaving 344 participants eligible for this analysis; 187 of these had their morphology scans performed at the Mercy Hospital for Women, and the remaining 157 were performed externally. Of the 344 participants with complete ultrasound data, 39 (11.3%) subsequently gave birth to SGA infants and were excluded from the main analysis. Growth velocities from the mid-trimester were therefore evaluated for a total of 305 fetuses subsequently delivered AGA (birthweight > 10th centile; Fig. 1). When we compared the external morphology ultrasound scans to those performed at the Mercy Hospital for women, there were no significant differences between the groups in gestation at time of morphology scan nor in customised EFW centile or AC centile (Additional file 1: Table S1).

### Twenty- to 36-week EFW and AC growth velocities

#### Antenatal evidence of placental insufficiency and 20–36-week growth velocity

**Fig. 2:**
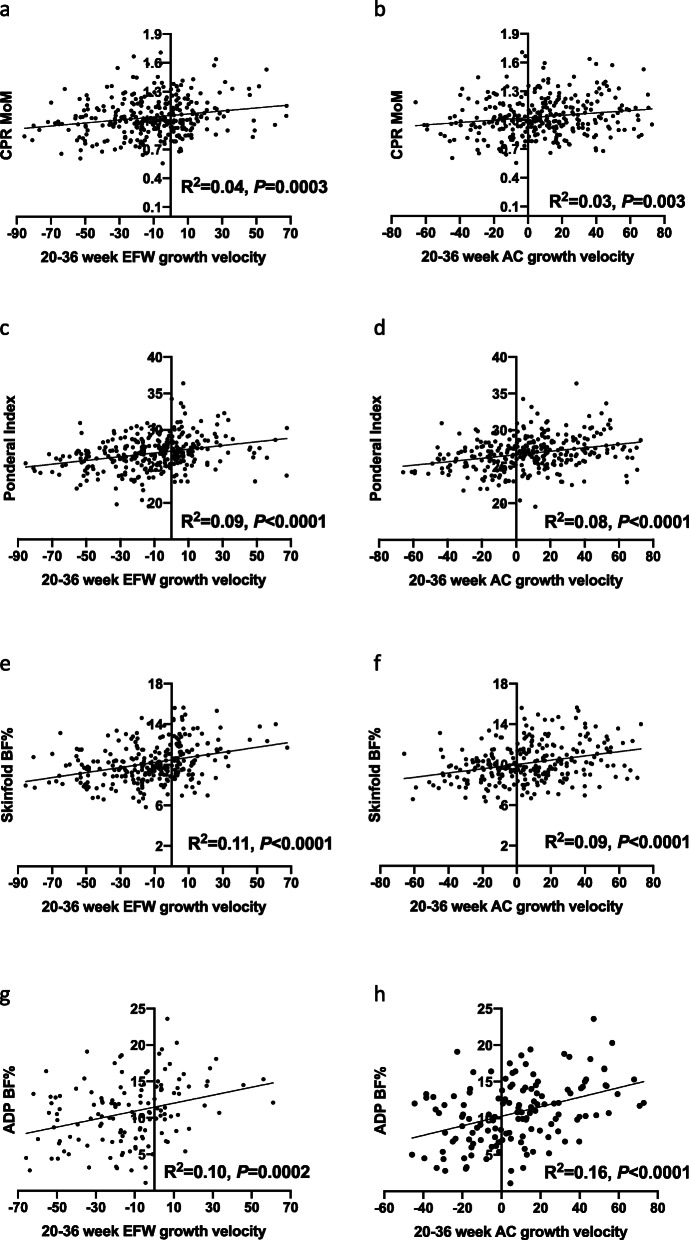
Twenty- to 36-week EFW and AC growth velocities and measures of placental insufficiency linear regression results. Cerebroplacental ratio (CPR) multiples of the median (MoM) according to **a** 20–36-week estimated fetal weight (EFW) growth velocity; **b** 20–36-week abdominal circumference (AC) growth velocity. Ponderal Index according to **c** 20–36-week EFW growth velocity; **d** 20–36 week AC growth velocity. Neonatal body fat percentage (BF%) as estimated by skinfold measurements according to **e** 20–36-week EFW growth velocity; **f** 20–36 week AC growth velocity. Neonatal BF% as estimated by air displacement plethysmography (ADP) according to **g** 20–36-week EFW growth velocity; **h** 20–36 week AC growth velocity

Both 20–36-week EFW and 20–36 week AC growth velocities positively correlated with CPR MoM (*P* = 0.0003 and 0.003 respectively; Fig. 2a, b). This suggests that the lower the EFW or AC growth velocity between 20 and 36 weeks, the lower the CPR, a marker fetal adaptation to placental insufficiency [18]. Thus, the lower the growth velocity between 20 and 36 weeks’ gestation, the greater the degree of fetal cerebral redistribution.

Using logistic regression, we then examined the relationship between 20 and 36-week EFW and AC growth velocities among AGA fetuses and low 36-week CPR (< 5th centile [23]) as a dichotomous outcome. This analysis was to investigate whether slowed growth velocities were associated with clinically significant cerebral redistribution, as this is a clinically recognised cut-off. Slowing 20–36-week EFW growth velocity was significantly associated with low CPR at 36 weeks’ gestation. For every one centile decrease in EFW velocity, the odds of low CPR at 36 weeks increased by 2.5% (95% confidence interval (CI) 0.9–4.0%, *P* = 0.001). The relationship between 20 and 36-week AC velocity and low 36-week CPR did not reach statistical significance (Table 1).

**Table 1:** Odds of indicators of placental insufficiency after univariate logistic regression, per centile decrease in EFW or AC between 20 and 36 weeks, and 20–28 weeks, compared to previously published odds per centile decrease in EFW or AC between 28 and 36 weeks [11]

Outcome	Growth parameter	Odds ratio (95% CI) of outcome per centile decrease from:					
		20–36 weeks	***P***	20–28 weeks	***P***	28–36 weeks	***P***
CPR < 5th centile [12] (*n* = 302)	EFW	1.025 (1.009–1.040)	0.001	1.013 (0.996–1.031)	0.13	1.024 (1.005–1.042)	0.01
	AC	1.014 (0.999–1.028)	0.07	1.005 (0.989–1.022)	0.52	1.015 (0.997–1.032)	0.10
Umbilical artery pH < 7.15 at birth (*n* = 238)	EFW	1.027 (1.009–1.045)	0.002	1.017 (0.996–1.038)	0.11	1.024 (1.003–1.045)	0.02
	AC	1.031 (1.013–1.048)	0.0005	1.021 (1.002–1.041)	0.03	1.022 (1.002–1.041)	0.03
ADP low body fat percentage [13] (*n* = 136)	EFW	1.016 (0.991–1.041)	0.22	0.995 (0.968–1.023)	0.74	1.033 (1.001–1.067)	0.047
	AC	1.028 (1.001–1.056)	0.04	1.005 (0.980–1.030)	0.70	1.036 (1.005–1.068)	0.02

*CI* confidence interval, *EFW* estimated fetal weight, *AC* abdominal circumference, *CPR* cerebroplacental ratio, *ADP* air displacement plethysmography. Participant numbers included for each outcome refer to those contributing to the 20–28- and 20–36-week growth velocity analyses

We next examined 20–36 EFW growth velocity at a relevant clinical threshold of − 30 centiles for detecting low CPR. We aimed to determine if a fall in EFW centile of this magnitude significantly elevated the risk of cerebral redistribution. Low CPR was significantly more common in fetuses that dropped over 30 EFW centiles across the 16-week period, with a RR of 2.2 (95% CI 1.1–4.4, *P* = 0.03) (Table 2).

**Table 2:** Relative risk of indicators of placental insufficiency when a 20–36-week EFW and AC growth velocity cut-off of < − 30 centiles is used to dichotomise the cohort

	Growth parameter	Low growth velocity ***n*** (%)	Growth velocity not low ***n*** (%)	RR (95% CI) if low 20–36-week growth velocity of < − 30 centiles	***P***
**CPR < 5th centile** [12]	EFW	11/65 (16.9)	18/237 (7.6)	2.23 (1.11–4.36)	0.03
**Umbilical artery pH < 7.15 at birth**	EFW	9/48 (18.8)	15/190 (7.9)	2.38 (1.11–4.93)	0.03
	AC	5/18 (27.8)	19/220 (8.6)	3.22 (1.32–6.93)	0.02
**ADP low body fat percentage** [13]	AC	1/9 (11.1)	11/127 (8.7)	1.28 (0.22–5.87)	0.58
**Placental weight < 10th centile** [21]	EFW	27/61 (44.3)	36/216 (16.7)	2.66 (1.75–3.96)	< 0.0001
	AC	10/22 (45.5)	53/255 (20.8)	2.19 (1.24–3.44)	0.01

*RR* relative risk, *CI* confidence interval, *EFW* estimated fetal weight, *AC* abdominal circumference, *CPR* cerebroplacental ratio, *ADP* air displacement plethysmography

Collectively, these results suggest that reduced EFW growth velocity between 20 and 36 weeks is significantly associated with cerebral redistribution at 36 weeks—an antenatal indicator of placental insufficiency.

Given there was a significant increase in risk when we dichotomised the cohort according to EFW 20–36-week growth velocity of < − 30 centiles or not, we compared the baseline maternal characteristics and pregnancy outcomes between these two groups. There were no significant differences in maternal characteristics, except that women with cases of low fetal growth velocity had statistically significantly lower median body mass index at booking. When it came to pregnancy outcomes, unsurprisingly, the low growth velocity group delivered significantly smaller infants, despite their AGA status, and their infants were also delivered at slightly earlier gestations (Table 3).

**Table 3:** Maternal characteristics and delivery outcomes of participants overall and comparison between low growth velocity (20–36-week EFW velocity < −30 centiles) and the remainder of the cohort

	Total analysis cohort (***n*** = 305)	Low growth velocity (***n*** = 67)	Normal growth velocity (***n*** = 238)	***P***
**Age (years)**	30.9 (4.2)	31.1 (4.2)	30.9 (4.2)	0.70
**Booking BMI (kg/m**^**2**^**)**	23.7 (21.5–26.9)	22.4 (20.9–26.2)	23.9 (21.8–27.1)	0.02
**Current smokers**	5 (1.6%)	1 (1.5%)	4 (1.7%)	1.00
**Gestational diabetes**	37 (12.1%)	10 (14.9%)	27 (11.3%)	0.40
**Onset of delivery**				
Spontaneous labour	136 (44.6%)	31 (46.3%)	105 (44.1%)	0.62
Induction labour	147 (48.2%)	33 (49.3%)	114 (47.9%)	
No labour	22 (7.2%)	3 (4.5%)	19 (8.0%)	
**Mode of delivery**				
Normal vaginal delivery	115 (37.7%)	32 (47.8%)	83 (34.9%)	0.15
Instrumental delivery	100 (32.8%)	22 (32.8%)	78 (32.8%)	
Emergency caesarean section	70 (23.0%)	10 (14.9%)	60 (25.2%)	
Elective caesarean section	20 (6.6%)	3 (4.5%)	17 (7.1%)	
**GA at delivery (weeks)**	40.0 (38.9–40.6)	39.7 (38.4–40.4)	40.0 (39.0–40.7)	0.04
**Birthweight (g)**	3453 (431.6)	3135 (312.3)	3543 (418.5)	< 0.0001
**Birthweight centile**	49.8 (26.7–71.8)	27.8 (15.8–36.4)	58.2 (32.4–77.5)	< 0.0001

Data presented as mean (standard deviation) or median (interquartile range) depending on distribution for continuous variables and as number (%) for categorical variables

Some percentages do not sum to 100% due to rounding to one decimal place

*BMI* body mass index, *GA* gestational age

Because of the significant difference seen in booking body mass index between the two groups, we next performed multivariate logistic regression to analyse the odds of low 36-week CPR with reducing 20–36-week EFW centile, accounting for maternal booking body mass index as a potential confounder. Maternal booking body mass index had no meaningful impact on the results. For every one centile decrease in 20–36-week EFW velocity, the adjusted odds of low CPR at 36 weeks increased by 2.6% (95% CI 1.0–4.1%, *P* = 0.001).

#### Intrapartum evidence of placental insufficiency and 20–36-week growth velocity

Intrapartum acidosis is a recognised clinical indicator of placental insufficiency. In response to the hypoxic challenge of labour, fetuses with less placental reserve are more likely to develop acidosis [19]. We investigated the relationship between reduced 20–36-week growth velocities and intrapartum acidosis (umbilical artery pH < 7.15 after labour) with logistic regression. Low EFW and AC 20–36-week growth velocities were both significantly associated with intrapartum acidosis. For every one centile decrease in EFW velocity, the odds of acidosis increased by 2.7% (95% CI 0.9–4.5%, *P* = 0.002). For every one centile decrease in AC velocity, the odds of acidosis increased by 3.1% (95% CI 1.3–4.8%, *P* = 0.0005) (Table 1).

We then performed multivariate logistic regression to assess 20–36-week fetal growth velocities and the odds of intrapartum acidosis accounting for maternal booking body mass index and gestational age at birth as potential confounders. Reducing EFW and AC 20–36-week growth velocities remained significantly associated with intrapartum acidosis. For every one centile decrease in EFW velocity, the adjusted odds of acidosis increased by 2.8% (95% CI 0.9–4.7%, *P* = 0.003). For every one centile decrease in AC velocity, the adjusted odds of acidosis increased by 3.2% (95% CI 1.3–5.0%, *P* = 0.001).

We next assessed the rates of intrapartum acidosis when the cohort was dichotomised as to whether the fetus had low EFW or AC growth velocities of < − 30 centiles between 20 and 36 weeks or not. Intrapartum acidosis was significantly more common for fetuses losing more than 30 EFW or AC centiles with RRs of 2.4 (95% CI 1.1–4.9, *P* = 0.03) and 3.2 (95% CI 1.3–6.9, *P* = 0.02) respectively (Table 2). Together, these results suggest that reduced fetal growth velocity between 20 and 36 weeks, demonstrated by falling EFW or AC centile, is associated with neonatal acidosis after labour—an intrapartum indicator of placental insufficiency.

#### Postnatal outcomes of placental insufficiency and 20–36-week growth velocity

Of the 305 infants, all had their Ponderal Index calculated, 268 (88%) had skinfold measurements performed and 136 (45%) had BF% estimated by air displacement plethysmography (ADP). We first performed linear regression to assess correlations between 20- and 36-week EFW and AC growth velocities and the continuous neonatal body composition measures. Both 20–36-week EFW and AC growth velocities were positively correlated with all three measures—Ponderal Index, skinfold BF% and ADP BF% (Fig. 2c–h). This suggests that the lower the EFW or AC growth velocity between 20 and 36 weeks, the lower the neonatal body fat—reflecting reduced nutrient provision while in utero.

We next investigated the relationship between 20- and 36-week growth velocities among AGA fetuses and abnormally low neonatal ADP BF% [20] with logistic regression (Table 1). Reduced AC 20–36-week growth velocity was significantly associated with low neonatal BF%, but 20–36 EFW velocity was not. For every one centile decrease in AC velocity, the odds of low neonatal BF% increased by 2.8% (95% CI 0.1–5.6%, *P* = 0.04). We only had 12 cases of low neonatal BF% in our cohort. This figure was too low to support a multivariate logistic regression model adjusting for maternal booking body mass index and gestation at birth. Instead, we assessed the individual effects of these potential confounders on the odds of low neonatal BF% with univariate logistic regression. Neither maternal booking BMI nor gestation at birth significantly increased the odds of low neonatal BF% (*P* = 0.06 and 0.16 respectively).

We then assessed the rates of low BF% when the cohort was dichotomised as to whether the fetus had low AC growth velocity of < − 30 centiles between 20 and 36 weeks. This clinical threshold of low AC growth velocity was not associated with a statistically significant increased risk of low BF% (Table 2), but this may be partly attributed to the smaller number of infants who had their BF% estimated by ADP. The results of the linear regression and logistic regression analyses together suggest that reduced fetal growth velocity between 20 and 36 weeks is associated with lower levels of neonatal body fat—postnatal evidence of placental insufficiency.

Placental weights were obtained for 277/305 (91%) of participants. When univariate logistic regression was performed, low EFW and AC 20–36-week growth velocities were both significantly associated with low placental weights of < 10th centile (adjusted for infant sex and gestation) [21]. For every one centile decrease in EFW and AC velocity, the odds of placental weight < 10th centile increased by 3.0% (95% CI 1.7–4.2%, *P* < 0.0001) and 2.1% (95% CI 0.9–3.2%, *P* = 0.0004) respectively.

Finally, we assessed the rates of placental weight < 10th centile when the cohort was dichotomised as to whether the fetus had low EFW or AC growth velocities of < − 30 centiles between 20 and 36 weeks or not. Placental weight of < 10th centile [21] was significantly more common for fetuses losing more than 30 EFW or AC centiles with RRs of 2.7 (95% CI 1.8–4.0, *P* < 0.0001) and 2.2 (95% CI 1.2–3.4, *P* = 0.01) respectively (Table 2). Together, these results suggest that reduced fetal growth velocity between 20 and 36 weeks, demonstrated by falling EFW or AC centile, is associated with small placentas, < 10th centile—a finding more common in cases of placental insufficiency.

#### Twenty- to 36-week growth velocity and indicators of placental insufficiency among the whole cohort

We also performed logistic regression to assess the relationships between EFW and AC growth velocities and indicators of placental insufficiency among the whole cohort (Additional file 2: Table S2). Because SGA infants (more likely to have experienced placental insufficiency) were not excluded from this analysis, we expected the associations between growth velocity and placental insufficiency indicators to be similar or stronger than the analyses performed among only the AGA. With SGA infants included, the relationships between fetal growth velocities and low 36-week CPR became stronger, and those with neonatal acidosis and low BF% were largely unchanged. In particular, there was a significant association demonstrated between 20- and 36-week AC growth velocity and low 36-week CPR (OR = 1.019, *P* = 0.003) when this did not reach statistical significance among the AGA only.

Because GROW customised centiles can only be calculated from 20 weeks’ gestation, we also analysed EFW growth velocity and indicators of placental insufficiency among the whole cohort if Hadlock centiles were used instead. The relationships seen between declining Hadlock EFW centiles and increased odds of the outcome measures were extremely similar to those demonstrated by declining customised EFW centiles (Additional file 2: Table S2).

### Twenty- to 28-week EFW and AC growth velocities

**Fig. 3:**
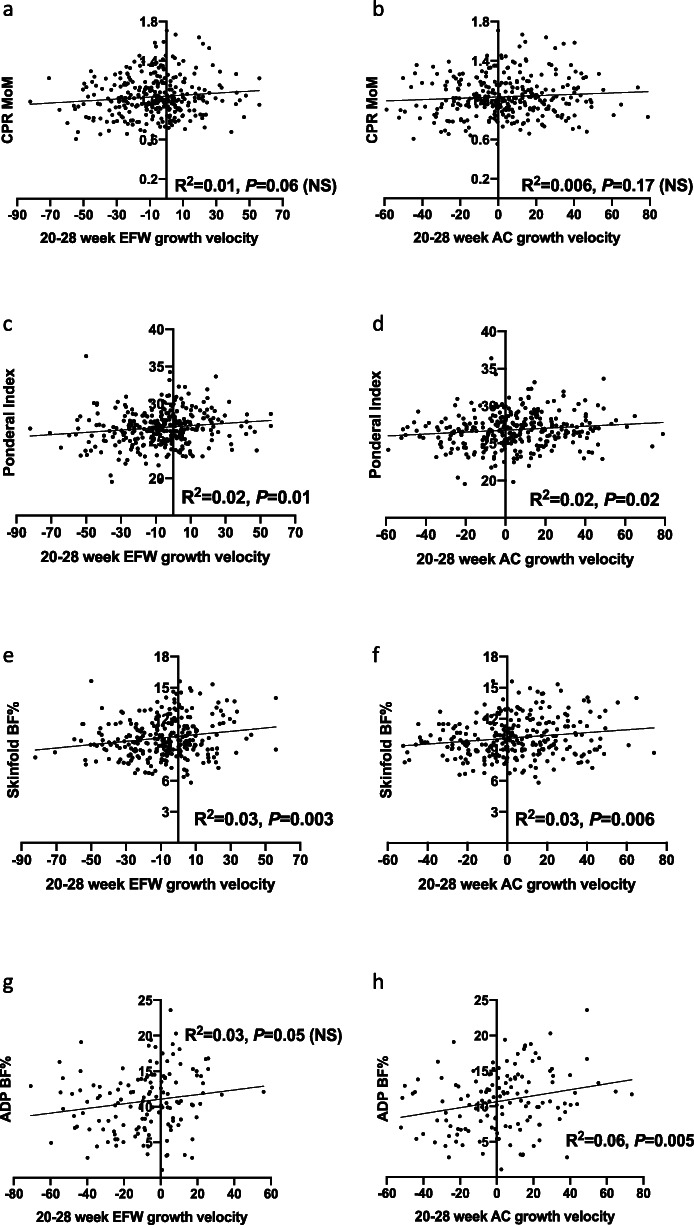
Twenty- to 28-week EFW and AC growth velocities and measures of placental insufficiency linear regression results. Cerebroplacental ratio (CPR) multiples of the median (MoM) according to **a** 20–28-week estimated fetal weight (EFW) growth velocity; **b** 20–28-week abdominal circumference (AC) growth velocity. Ponderal Index according to **c** 20–28-week EFW growth velocity; **d** 20–28-week AC growth velocity. Neonatal body fat percentage (BF%) as estimated by skinfold measurements according to **e** 20–28-week EFW growth velocity; **f** 20–28-week AC growth velocity. Neonatal BF% as estimated by air displacement plethysmography (ADP) according to **g** 20–28-week EFW growth velocity; (**h**) 20–28-week AC growth velocity

We also examined whether EFW and AC growth velocities between 20 and 28 weeks were associated with clinical indicators of placental insufficiency. When linear regression was performed, 20–28-week growth velocities were not significantly correlated with CPR MoM (Fig. 3a, b). EFW and AC 20–28-week growth velocities positively correlated with all neonatal body fat measures—Ponderal Index, neonatal BF% estimated by skinfold testing and ADP BF%, except 20–28-week AC growth velocity and ADP BF% (Fig. 3c–h), but with weaker correlations than those demonstrated by 20–36-week growth velocities. Using logistic regression, we did not identify significant relationships between 20- and 28-week EFW and AC growth velocities and any of the antenatal, intrapartum or neonatal indicators of placental insufficiency (Table 1) except for 20–28 week AC growth velocity and intrapartum acidosis. For every one centile decrease in AC velocity, the odds of intrapartum acidosis increased by 2.1% (95% CI 0.2–4.1%, *P* = 0.03). However, when we dichotomised the cohort, low growth velocity defined as 20–28 week AC velocity of < − 30 centiles was not associated with significantly increased risk of intrapartum acidosis (RR (95% CI) = 1.5 (0.4–4.5), *P* = 0.64).

## Discussion

### Main findings

We report that reduced EFW and AC growth velocities across the gestational epoch of 20–36 weeks are associated with antenatal, intrapartum and neonatal indicators of placental insufficiency in infants born AGA. We found significant correlations between reduced 20–36-week growth velocities and (i) low CPR at 36 weeks’ gestation, reflective of adaptive fetal cerebral redistribution and increased placental resistance; (ii) development of acidosis under the hypoxic challenge of labour; (iii) low neonatal body fat stores; and (iv) small placentas with weight < 10th centile for infant sex and gestation, even though the majority of fetuses with low growth velocity did not exhibit these features of placental insufficiency. This supports our previous findings suggesting that AGA fetuses with reduced growth velocity across the third trimester may have experienced placental insufficiency, placing them at increased risk of stillbirth [11, 24] despite their ‘normal’ birthweight. This study reports that the routine mid-trimester ultrasound can be used as the baseline assessment of fetal size from which a reliable measure of slowing growth can be generated at 36 weeks’ gestation. Slowing of growth across this gestational epoch is associated with features of placental insufficiency at term.

Importantly, these findings suggest this novel assessment of fetal wellbeing could be feasibly achieved with the addition of just a single 36-week scan to standard antenatal care. We report that fetal growth velocity between 20 and 36 weeks compares favourably to our previously reported associations of 28–36-week fetal growth velocity and features of placental insufficiency [11], potentially obviating the need for an additional 28-week ultrasound. Furthermore, this adds to the previously established benefits of a routine 36-week scan: increased detection of SGA, large-for-gestational-age and breech presentation at term [12, 25–28]. Although a large randomised controlled trial of routine third trimester ultrasound did not demonstrate a reduction in severe adverse perinatal outcomes [28], the additional information provided from fetal growth velocity assessment may add value to the 36-week scan. Identifying AGA fetuses with slowed growth velocity may unmask a currently unrecognised group experiencing placental insufficiency, who are potentially at increased risk of stillbirth.

We did not demonstrate strong relationships between 20- and 28-week growth velocities and indicators of placental insufficiency at term. This finding is in keeping with the hypothesised pathophysiology of late-onset FGR which typically occurs at or beyond 32 weeks’ gestation [9, 29]. Placental insufficiency occurring early enough in pregnancy to cause growth decrements during the mid-trimester would likely culminate in the birth of an infant preterm, SGA or both. Such infants were excluded from our analyses given they are already known to represent high-risk cohorts.

### Interpretation of the results and comparison with other studies

This study supports the findings of the Pregnancy Outcome Prediction study, where low 20–36-week AC growth velocity was associated with poorer perinatal outcomes among fetuses with an EFW < 10th centile at 36 weeks [12]. In the Pregnancy Outcome Prediction study, SGA fetuses with AC growth velocity in the lowest decile had a RR of 2.5 for neonatal morbidity, compared to a RR of only 1.3 for SGA fetuses who had exhibited normal AC growth velocity from the mid-trimester. Similar associations were observed for other adverse outcomes, such as metabolic acidosis, neonatal unit admission and severe adverse perinatal outcome. This highlights the added value of fetal growth velocity assessment to size among SGA fetuses in late pregnancy.

Our findings suggest that 20–36-week growth velocity is also an important indicator of wellbeing among AGA fetuses. We have previously reported significant associations between 28- and 36-week growth velocity and antenatal, intrapartum and postnatal indicators of placental insufficiency [11] in a prospective cohort of low risk nulliparous women who delivered AGA infants. The current study demonstrates that these growth trajectory assessments could start from the time of routine mid-trimester ultrasound examination, given that 20–36-week fetal growth velocities predict indicators of placental insufficiency as well as those calculated between 28 and 36 weeks. A previous study of 934 AGA infants also reported significant inverse associations between abdominal circumference velocity from the mid-trimester and (i) composite adverse neonatal outcome and (ii) Neonatal Intensive Care admission [30]. An important limitation however was its retrospective design. The third trimester data came from clinically indicated referral ultrasounds, where the biometry was not blinded, and the results were used to inform clinical care. In contrast, our prospective study suggests that these findings may be generalisable to unselected populations.

### Strengths and limitations

Major strengths of this study include its prospective design and the examination of multiple, robust measures of placental insufficiency across the antenatal, intrapartum and neonatal periods. CPR, umbilical artery pH, ADP BF% and small placental size are each reliable indicators of placental insufficiency [19, 22, 31–33], and the relationships between growth velocity and these outcome measures were consistent. The study was also strengthened by our use of customised EFW and birthweight centiles, which have a stronger association with adverse perinatal outcomes than standard population references [34]. By calculating centile change per day, we were able to perform standardised comparisons. By then dichotomising the cohort according to a definition of low growth velocity (loss of 30 centiles between 20 and 36 weeks), we have aided the clinical interpretation, and potential application, of our data.

A limitation of the study is that we were unable to assess for intra-observer variability among fetal biometry measurements recorded at the mid-trimester morphology scans. Additionally, there was greater variability in the timing and locations of the mid-trimester ultrasound scans (17^+6^ to 23^+0^ weeks inclusive) when compared to the standardised 28- and 36-week research scans (27^+0^ to 29^+0^ and 35^+0^ to 37^+0^ respectively). Despite the greater heterogeneity of the mid-trimester scans, the relationships observed between growth velocities measured from 20 to 36 weeks, versus 28–36 weeks, and indicators of placental insufficiency were remarkably similar (Table 1). That the mid-trimester morphology scans were performed at more variable sites and gestations reflects real-life clinical practice and potentially increases the generalisability of our findings. In addition, there were no significant differences in the median gestations of the mid-trimester scans, nor the AC or EFW centiles derived from these depending on whether they were performed internally or by external providers (Additional file 1: Table S1). That we were unable to perform histopathology on the placentas of study participants is a further limitation. Finally, the study was limited by its cohort size. It was underpowered to detect important but uncommon perinatal outcomes such as stillbirth. Results from this study warrant validation in a larger study.

### Clinical and research implications

This study suggests that calculation of fetal growth velocity between the routine mid-trimester ultrasound and 36 weeks could help identify undetected placental insufficiency among AGA fetuses. It is plausible that these fetuses may represent an unsuspected cohort at increased risk of stillbirth. Fetuses with low growth velocity may benefit from increased surveillance and timely delivery to reduce the risks associated with placental insufficiency, including stillbirth. This study suggests that—with the addition of only one 36-week scan to routine antenatal care—identification of FGR among the AGA could be increased. Given that universal late-pregnancy ultrasound has already been established to (i) almost triple the identification of SGA, (ii) identify which SGA fetuses are at greatest risk of adverse outcome, (iii) almost eliminate undiagnosed breech presentation and its associated mortality and (iv) be cost effective [12, 26, 35], this additional assessment could be feasibly incorporated into clinical care. Secondly, the increases in risk reported here associated with loss of greater than 30 or more EFW or AC centiles may assist clinicians interpreting fetal growth velocity when caring for pregnant women who have undergone serial ultrasounds scans where the fetus is not—or not yet—SGA. Here, we have demonstrated the feasibility of generating growth velocity per day values. From these, a fixed multiplier can be used to standardise comparison between scans, irrespective of the exact gestations at which they are performed. Finally, by identifying that the baseline assessment to calculate fetal growth velocity can be undertaken as early as the mid-trimester, this study proposes a novel application of the routine morphology ultrasound.

These findings suggest that a fall of 30 or more EFW or AC centiles between the 20- and 36-week scans could be used as a pragmatic clinical cut-off to define low fetal growth velocity, given the consistent associations with markers of placental insufficiency. Future research will involve testing this threshold in a larger prospective study to more comprehensively determine its diagnostic performance for adverse perinatal outcomes. If confirmed, AGA fetuses with reduced growth velocity may be candidates for increased surveillance and timely delivery to reduce stillbirth risk.

## Conclusions

Improved detection of FGR among AGA fetuses is an unmet gap in current clinical care. AGA infants demonstrating reduced fetal growth velocities from as early as the mid-trimester exhibit antenatal, intrapartum and neonatal indicators of placental insufficiency typically associated with growth restricted infants. EFW and AC fetal growth velocities between 20 and 36 weeks’ gestation perform similarly to previously described 28–36-week growth velocities in predicting measures of placental insufficiency. This additional assessment of fetal wellbeing can thus be obtained from adding only one ultrasound scan at 36 weeks to routine care. If these findings are validated in larger studies, AGA fetuses with reduced growth velocity may represent a previously unrecognised cohort who could benefit from increased surveillance and timely delivery to reduce late pregnancy stillbirth risk.

## Supplementary Information

**Additional file 1: Table S1**. Comparison of mid-trimester morphology ultrasound scans performed internally at the Mercy Hospital for Women to those performed externally.

**Additional file 2: Table S2**. Odds of indicators of placental insufficiency among the whole cohort after univariate logistic regression, per centile decrease in customised EFW, Hadlock EFW, or AC between 20 and 36 weeks.

## Data Availability

The datasets generated and analysed during the current study are available from the corresponding author on reasonable request.

## Abbreviations

AC: Abdominal circumference
ADP: Air displacement plethysmography
AGA: Appropriate-for-gestational-age
BF%: Body fat percentage
CI: Confidence interval
CPR: Cerebroplacental ratio
EFW: Estimated fetal weight
FGR: Fetal growth restriction
FLAG: Fetal Longitudinal Assessment of Growth
MoM: Multiples of the median
OR: Odds ratio
RR: Relative risk
SGA: Small-for-gestational-age
