# Supplementary material for: Reduced growth velocity from the mid-trimester is associated with placental insufficiency in fetuses born at a normal birthweight
Source: BMC Med. 2020 Dec 24;18:395. doi: 10.1186/s12916-020-01869-3 (PMC7758928; doi:10.1186/s12916-020-01869-3)
Supplement: Supplementary file 1 — Additional file 1: Table S1. Comparison of mid-trimester morphology ultrasound scans performed internally at the Mercy Hospital for Women to those performed externally. [file 12916_2020_1869_MOESM1_ESM.docx]

**Table S1: *Comparison of mid-trimester morphology ultrasound scans performed internally at the Mercy Hospital for Women to those performed externally***

| **Ultrasound characteristic** | **Internally performed** | **Externally performed** | ***P*** |
| --- | --- | --- | --- |
| **Gestation at scan (weeks)** | 20.0 (19.7-20.4) | 20.1 (19.8-20.6) | 0.09 |
| **Customised EFW centile** | 80.2 (66.9-91.6) | 75.7 (57.3-93.4) | 0.24 |
| **AC centile** | 56.9 (39.9-77.8) | 57.6 (37.4-71.6) | 0.30 |

Data presented as median (interquartile range). AC = abdominal circumference, EFW = estimated fetal weight.
