# Supplementary material for: Reduced growth velocity from the mid-trimester is associated with placental insufficiency in fetuses born at a normal birthweight
Source: BMC Med. 2020 Dec 24;18:395. doi: 10.1186/s12916-020-01869-3 (PMC7758928; doi:10.1186/s12916-020-01869-3)
Supplement: Supplementary file 2 — Additional file 2: Table S2. Odds of indicators of placental insufficiency among the whole cohort after univariate logistic regression, per centile decrease in customised EFW, Hadlock EFW, or AC between 20 and 36 weeks. [file 12916_2020_1869_MOESM2_ESM.docx]

**Table S2: *Odds of indicators of placental insufficiency among the whole cohort after univariate logistic regression, per centile decrease in customised EFW, Hadlock EFW, or AC between 20-36 weeks***

| **Outcome** | **Growth Parameter** | **Odds Ratio (95% CI) of outcome per centile decrease 20-36 weeks** | ***P*** |
| --- | --- | --- | --- |
| CPR <5^th^ Centile (12) | EFW - customised (n = 341) | 1.027 (1.014-1.039) | <0.0001 |
|  | EFW - Hadlock (n = 339) | 1.026 (1.013-1.040) | 0.0001 |
|  | AC (n = 341) | 1.019 (1.006-1.031) | 0.003 |
| Umbilical artery pH <7.15 at birth | EFW – customised (n = 267) | 1.023 (1.008-1.038) | 0.003 |
|  | EFW – Hadlock (n = 265) | 1.024 (1.008-1.040) | 0.003 |
|  | AC (n = 267) | 1.027 (1.012-1.042) | 0.0004 |
| ADP low body fat percentage (13) (n = 151) | EFW – customised | 1.016 (0.995-1.038) | 0.14 |
|  | EFW – Hadlock | 1.022 (0.998-1.045) | 0.07 |
|  | AC | 1.024 (1.001-1.047) | 0.04 |

In these analyses, small-for-gestational-age infants (birthweight <10^th^ centile) remain included (as opposed to the data presented in Table 1). CI confidence interval, EFW = estimated fetal weight, AC = abdominal circumference, CPR cerebroplacental ratio, ADP air displacement plethysmography.
